# CMR provides comparable measurements of diastolic function as echocardiography

**DOI:** 10.1038/s41598-024-61992-6

**Published:** 2024-05-22

**Authors:** Kana Fujikura, Bharath Sathya, Tushar Acharya, Mitchel Benovoy, Matthew Jacobs, Vandana Sachdev, Li-Yueh Hsu, Andrew E. Arai

**Affiliations:** 1 Department of Health and Human Services, National Heart, Lung and Blood Institute, National Institutes of Health, Bethesda, MD USA; 2 Radiology and Cardiology, NYU Grossman School of Medicine, New York, NY USA; 3 Cardiovascular Medicine and Department of Radiology, University of Utah, Salt Lake City, UT USA

**Keywords:** Cardiac magnetic resonance imaging, Echocardiography, Left ventricle, Diastolic function, Left atrial volume, Cardiology, Cardiovascular diseases

## Abstract

Clinical application of cardiac magnetic resonance (CMR) is expanding but CMR assessment of LV diastolic function is still being validated. The purpose of this study was to validate assessments of left ventricular (LV) diastolic dysfunction (DD) using CMR by comparing with transthoracic echocardiography (TTE) performed on the same day. Patients with suspected or diagnosed cardiomyopathy (n = 63) and healthy volunteers (n = 24) were prospectively recruited and included in the study. CMR diastolic parameters were measured on cine images and velocity-encoded phase contrast cine images and compared with corresponding parameters measured on TTE. A contextual correlation feature tracking method was developed to calculate the mitral annular velocity curve. LV DD was classified by CMR and TTE following 2016 guidelines. Overall DD classification was 78.1% concordant between CMR and TTE (*p* < 0.0001). The trans-mitral inflow parameters correlated well between the two modalities (E, r = 0.78; A, r = 0.90; E/A, r = 0.82; all *p* < 0.0001) while the remaining diastolic parameters showed moderate correlation (e’, r = 0.64; E/e’, r = 0.54; left atrial volume index (LAVi), r = 0.61; all *p* < 0.0001). Classification of LV diastolic function by CMR showed good concordance with standardized grades established for TTE. CMR-based LV diastolic function may be integrated in routine clinical practice.

*Name of the registry*: Technical Development of Cardiovascular Magnetic Resonance Imaging. Trial registration number: NCT00027170. Date of registration: November 26, 2001. URL of trial registry record: https://clinicaltrials.gov/ct2/show/NCT00027170

## Introduction

Evaluation of left ventricular (LV) diastolic dysfunction (DD) is an integral part of the routine cardiac assessment of individuals with shortness of breath or heart failure. In order to standardize the assessment of LV DD, the American Society of Echocardiography and European Association of Cardiovascular Imaging diastolic function working group developed guidelines^1^. Based on this collaborative effort, the Intersocietal Accreditation Commission has established a standard for echocardiography to incorporate assessment of LV DD as a part of routine clinical practice^2^.

LV DD is a result of increased LV chamber stiffness and impaired LV relaxation which subsequently elevates cardiac filling pressures. With echocardiography, LV DD can be assessed by a combination of various parameters including trans-mitral LV inflow using Doppler, motion of the mitral valve annulus acquired by tissue Doppler imaging (TDI), indexed size of the left atrium (LAVi) using 2-dimensional imaging, and pulmonary pressures estimated by TR^1^.

Cardiac magnetic resonance imaging (CMR) is a versatile modality that enables highly reproducible and accurate assessment of cardiac morphology and function. CMR provides unique tissue characterization of various myocardial disease processes^3,4^. Since diastolic function is important in many of these types of patients, it would be desirable to develop reliable methods to assess LV DD using CMR.

Cine CMR imaging can track the position of certain anatomical structures throughout the cardiac cycle^5^. Using this technique, we developed an automatic method to derive the maximum velocity of mitral valve annulus (MVA) using a spatiotemporal learning approach to track multichannel discriminative features of cardiac landmarks in CMR cine imaging.

Our primary goal of this study was to assess the feasibility of classifying LV DD using CMR compared with transthoracic echocardiogram (TTE). The secondary purpose was to evaluate the concordance and discordance of diastolic parameters measured CMR and TTE on the same individuals.

## Results

### Baseline cohort

**Figure 1:**
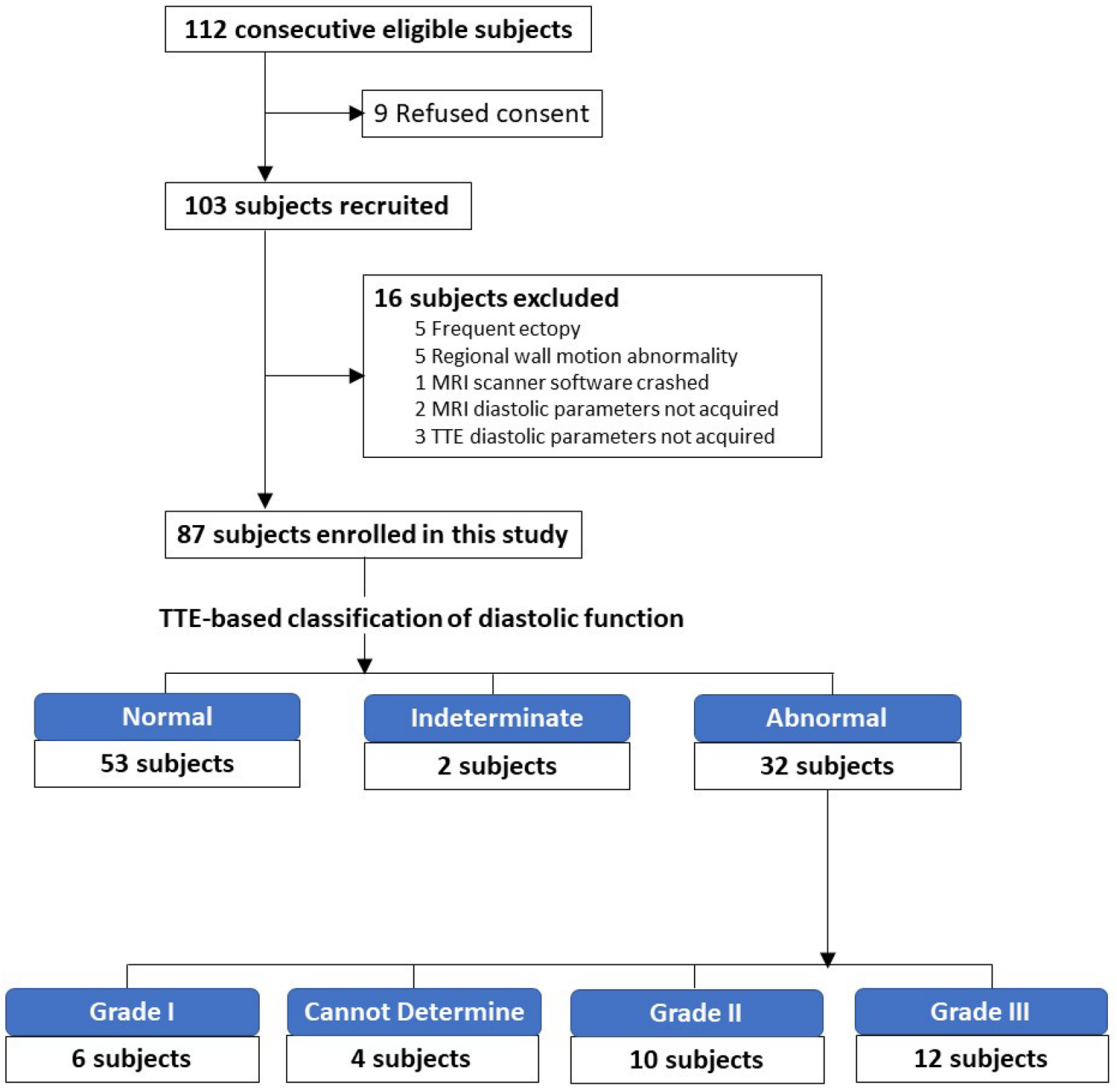
Patient recruitment and distribution of diastolic function. *MRI* magnetic resonance imaging; *TTE* transthoracic echocardiography.

112 subjects were eligible for this study, however 9 refused to sign the consent. Among the 103 subjects recruited for this study, 16 patients were excluded (Fig. 1). The final study population comprised 87 subjects and included 63 patients with suspected or diagnosed cardiomyopathy and 24 healthy volunteers. For the 63 patients with suspected or diagnosed cardiomyopathy, the reason for CMR was HCM (n = 28), infiltrative cardiomyopathy (n = 17), myocarditis (n = 8), other non-ischemic cardiomyopathy (n = 7), and atypical chest pain (n = 3). TTE and CMR were performed on the same day in 85 patients (97.7%); there was a 1-day and 9-day discrepancy between TTE and CMR studies due to scanner and scheduling issues in the other subjects.

### Characteristics of subjects

TTE-based LV DD divided the study subjects into a normal (n = 53), Indeterminate (n = 2), and an abnormal (n = 32) groups (Fig. 1), and characteristics of each group are summarized in Table 1. Subjects with abnormal LV diastolic function were older, more likely to have a history of hypertension, had higher blood pressure, and had larger body mass index compared with subjects with normal diastolic function.

**Table 1:** Baseline characteristics.

	Total (n = 87)	Normal or Indeterminate diastolic function (n = 53)	Abnormal diastolic function (n = 32)	
Male, n (%)	46 (56.8)	30 (54.6)	16 (61.5)	0.63
Age, y ± SD	45 ± 18	40 ± 18	55 ± 12	< 0.0001*
Smoking, n (%)	6 (7.6)	2 (3.7)	4 (16.0)	0.08
Hypertension, n (%)	25 (31.7)	10 (18.5)	15 (60.0)	0.0005*
Diabetes, n (%)	8 (10.1)	5 (9.3)	3 (12.0)	0.70
Coronary artery disease, n (%)	2 (2.6)	2 (3.7)	0 (0.0)	1.00
Hypertrophic cardiomyopathy, n (%)	13 (16.1)	0 (0.0)	13 (50.0)	< 0.0001*
Body surface area, m^2^ ± SD	1.9 ± 0.3	1.9 ± 0.2	2.0 ± 0.3	0.087
Systolic blood pressure, mmHg ± SD	130 ± 18	124 ± 13	142 ± 22	0.0007*
Diastolic blood pressure, mmHg ± SD	72 ± 11	70 ± 10	74 ± 12	0.09
Heart rate, beat per minute ± SD	65 ± 10	70 ± 12	69 ± 11	0.75

* *p* = 0.008.

### Correlation between CMR- and TTE-measurements (Figs. 2 and 3, and Supplemental Table 1)

On trans-mitral valve flow measurements, there were strong correlations in E, A, and E/A between CMR and TTE (r = 0.78, 0.90, 0.82, respectively; all *p* < 0.0001). Regarding annular motion velocity, averaged e’ showed a good correlation between CMR and TTE (r = 0.71, *p* < 0.0001). E/e’ showed moderate correlation between the two modalities (r = 0.58, *p* < 0.0001). Comparing the values between the two modalities, E, A, and E/e’ were significantly larger in CMR (*p* < 0.0001, *p* < 0.0001, and 0.0016, respectively), whereas E/A and average e’ were similar (p = 0.056 and 0.10, respectively). For LAVi, CMR measurements moderately correlated with TTE measurement (r = 0.61, *p* < 0.0001), however the values were significant larger with CMR (*p* < 0.0001).

**Figure 2:**
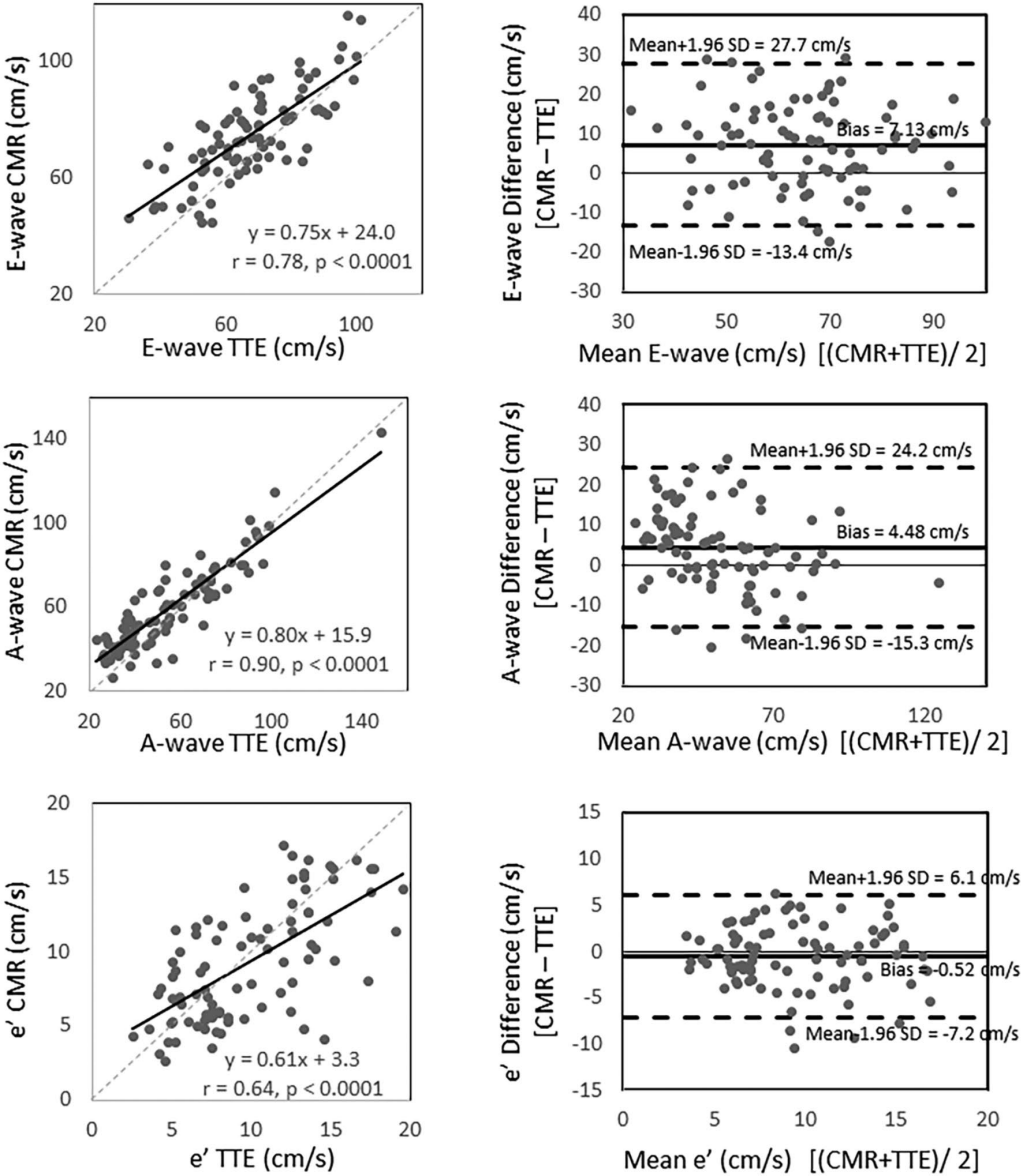
Linear regression (left) and Bland–Altman Plots (right) comparing CMR and TTE on E-wave (top), A-wave (middle), and average e’ (bottom). *A* late trans-mitral filling during diastole; *CMR* cardiac magnetic resonance; *E* early trans-mitral filing during diastole; *e’* maximum mitral-annulus motion velocity during early diastole; *TTE* transesophageal echocardiography.

**Figure 3:**
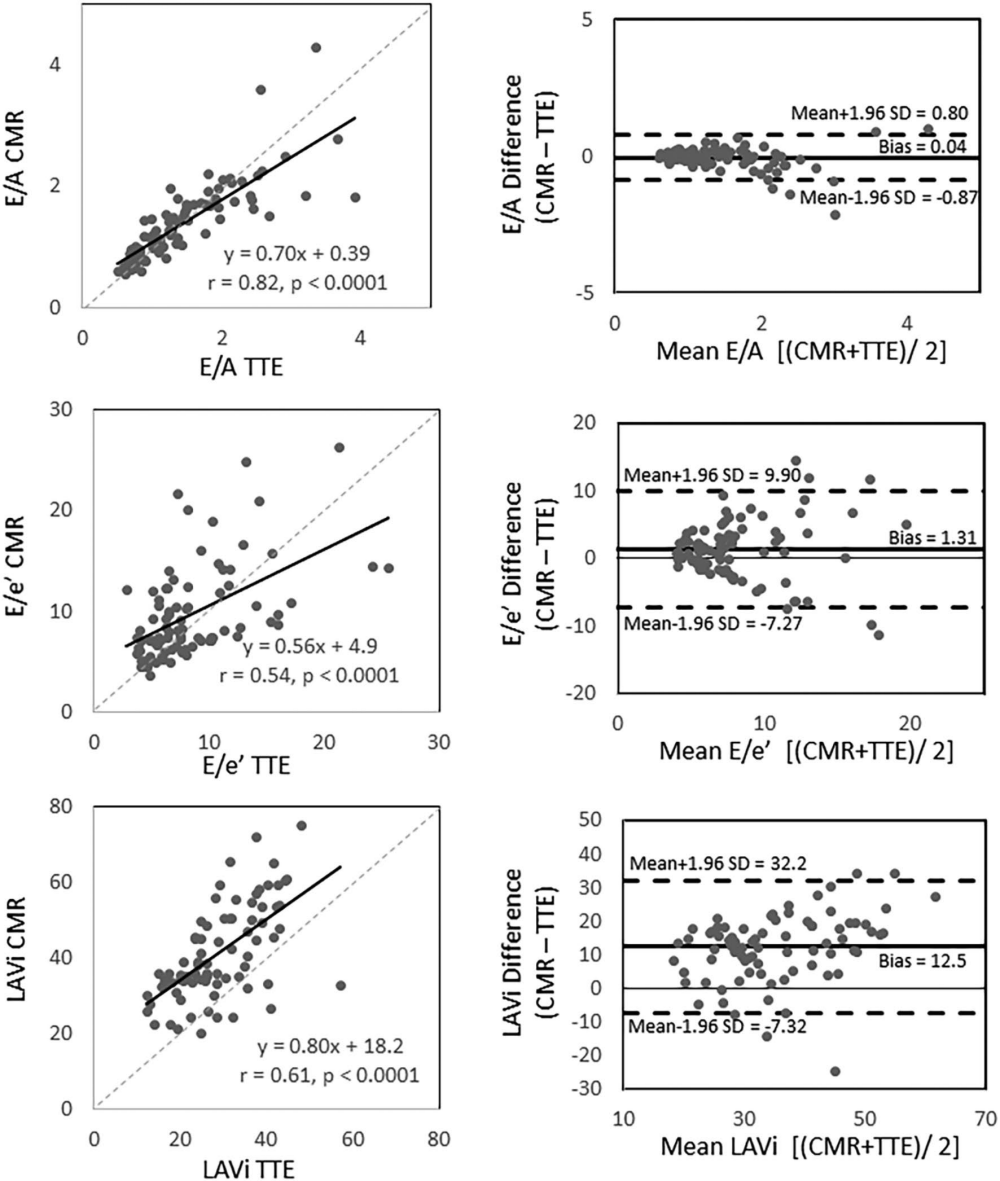
Linear regression (left) and Bland–Altman Plots (right) comparing CMR and TTE on E/A (top), E/e’ (middle), and LAVi (bottom). *CMR* cardiac magnetic resonance; *E/A* ratio of maximum trans-mitral flow velocity of the early- and late-filling during diastole; *E/e’* ratio of maximum trans-mitral flow velocity of the early trans-mitral filling during diastole and maximum mitral-annulus motion velocity during early diastole; *TTE* transesophageal echocardiography; *LAVi* left atrial volume index.

### Concordance grading severity of diastolic dysfunction by CMR and TTE

**Figure 4:**
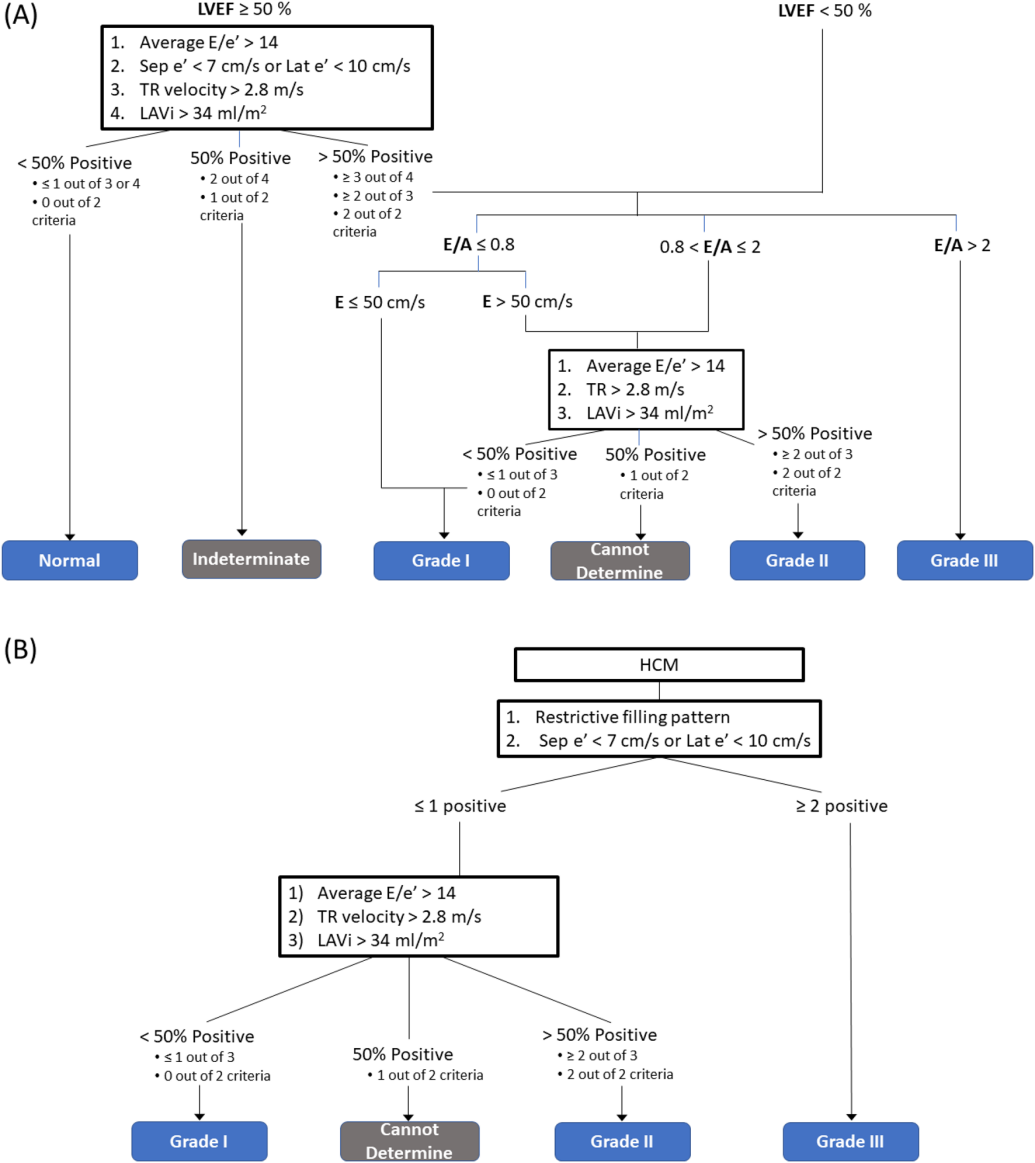
Diagram to classify LV diastolic function based on 2016 ASE Guidelines for (**A**) all the subjects except patients with HCM, and (**B**) patients with HCM. *A* late trans-mitral filling; *E* maximum velocity of early trans-mitral filling; *e’* average mitral valve annular velocity; *HCM* hypertrophic cardiomyopathy; *LAVi* left atrial volume index; *LV* left ventricle; *TR* tricuspid regurgitation.

Criteria for hypertrophic cardiomyopathy were present in 15 subjects by TTE (Table 2) and 18 subjects by CMR (Table 3) and thus had LV DD categorized using the algorithm described in Fig. 4B. All the other subjects were categorized using the algorithm described in Fig. 4A.

**Table 2:** LV diastolic function classification of subjects with and without HCM in echocardiography.

	LV diastolic dysfunction						Total
	Normal	Indeterminate	Grade I	Cannot determine	Grade II	Grade III	
No HCM	53	2	4	3	10	0	72
HCM	0	0	2	1	0	12	15
Total	53	2	6	4	10	12	87

*HCM* hypertrophic cardiomyopathy; *LV* left ventricle.

**Table 3:** LV diastolic function classification of subjects with and without HCM in CMR.

	LV diastolic dysfunction						Total
	Normal	Indeterminate	Grade I	Cannot determine	Grade II	Grade III	
No HCM	53	0	10	3	3	0	69
HCM	0	–	0	0	0	18	18
Total	53	0	10	3	3	18	87

*HCM* hypertrophic cardiomyopathy; *LV* left ventricle.

**Figure 5:**
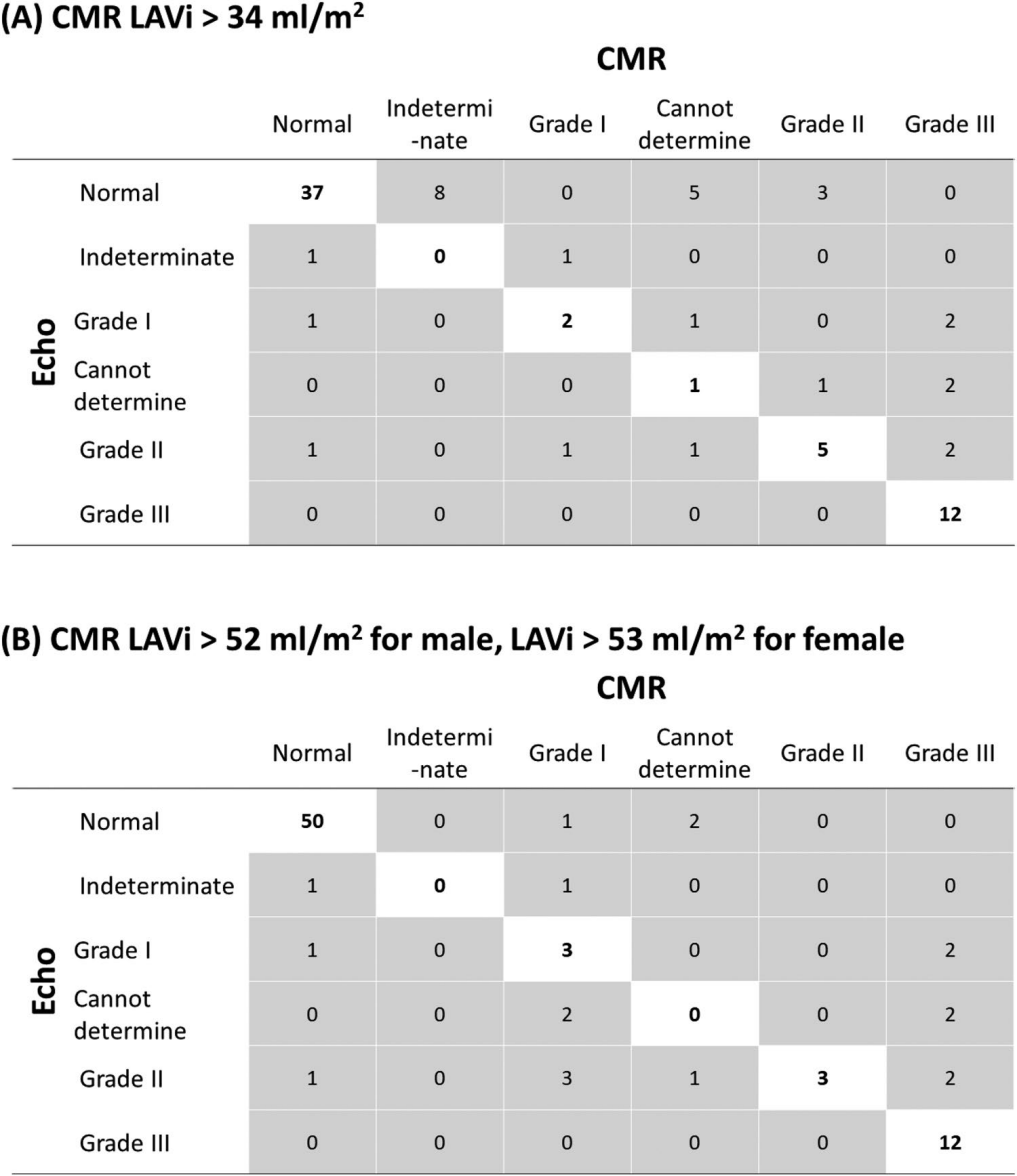
Comparing CMR- and TTE-based diastolic function classification. *CMR* cardiac magnetic resonance; *LAVi* left atrial volume index; *TTE* transesophageal echocardiography.

Based on the differences between LAVi measurements by TTE and CMR as described above, CMR-based LV DD assessment was performed using two different cut-off values of LAVi: 1) 52 ml/m^2^ for male and 53 ml/m^2^ for female based on reported normal LAVi values on CMR^6^, and 2) 34 ml/m^2^ which is the same value as TTE cut-off value. Overall the LV DD classification was statistically similar between CMR and TTE regardless of the cut-off values of LAVi (both *p* < 0.0001) (Fig. 5). However, the concordance of LV DD classification between the two modalities improved when CMR-based normal LAVi values were used rather than using the TTE cut-off values (12.6% improvement).

## Discussion

LV DD is pivotal information used to guide clinical management of patients with symptoms of heart failure. Assessment of LV DD requires measurements of trans-mitral blood flow and MVA velocities which are not a routine part of current clinical CMR protocols. Recently Ramos et al.^7^ measured these parameters using sector-wise golden-angle phase contrast sequence, a new method developed in their laboratory. In our paper, all of our CMR image acquisitions were performed using product level image acquisition sequences with parameters optimized to capture LV DD. Trans-mitral blood flow was acquired using through-plane velocity-encoded phase contrast imaging, and MVA velocity was analyzed using SSFP cine imaging. We present a rapid and reliable post-processing method to track MVA throughout a cardiac cycle on CMR SSFP cine imaging and subsequently derive e’. In this study, we obtained SSFP cine imaging with higher temporal resolution which is almost double compared to SSFP cine sequence used in daily clinical practice. Our CMR-based LV DD classification showed good agreement with TTE-based classification. Feasibility of high-quality diastolic measurements with Interactive Data Language (IDL)-based software suggests these developments can be quickly incorporated into clinical practice.

One of the distinctive designs of this prospective study was the same-day performance of CMR and TTE studies. Only two patients out of 87 had TTE on a different day from CMR, one had TTE the next day, and another had TTE 8 days after the CMR study. Compared to the recent study of Ramos et al.^7^ which comprised of 46 patients with 3 [0, 16] days separation between TTE and CMR, the current study may provide a better comparison between the two modalities.

Multiple parameters are affected by LV DD deterioration. E mitral annulus velocity is not sensitive to preload, and e’ stays reduced throughout all grades of LV DD^8^. Therefore e’ is one of the most important parameters in classifying LV DD. Measurement of e’ using CMR has been studied using phase-contrast and feature-tracking. Paelink et al.^9^ showed a strong correlation between CMR and TTE measurements in E/e’ using a relatively small cohort of 18 patients with hypertensive heart disease. Wu et al.^5^ created a semi-automated MVA tracking system on apical 2-chamber, 3-chamber, and 4-chamber cine-CMR images, and assessed mitral annulus sweep rate (i.e. rate of volume change within the path of mitral annulus during a cardiac cycle). Early-to-late diastolic annular velocity ratio (e’/a’) showed a good correlation between CMR and TTE TDI even though the images of the two modalities were acquired up to two months apart. Ramos et al.^7^ measured e’ on CMR using an experimental golden-angle sequence and showed a strong correlation with TTE-based measurements. In our study, SSFP was acquired using high temporal resolution with (13.8 ms per image, 60 phases per cardiac cycle) which is about twice as high as the standard clinical acquisitions (30–45 ms per image, 20–30 phases per cardiac cycle). However, our temporal resolution is still low compared to TTE TDI which is approximately 200 frames per second^10^. Regardless of the limited temporal resolution, our study showed comparable e’ values between CMR and TTE. One should carefully consider the Bland–Altman results. Inadequate temporal resolution by CMR should have caused a bias relative to TTE. This was not observed within the statistical power of the study. Bland–Altman results for e’ and E/e’ both show heteroscedasticity with increasing values. However, this scatter cannot be attributed to CMR or TTE without a comparison to a gold standard measurement.

Temporal resolution of CMR is also a challenge in the assessment of trans-mitral inflow (E and A on velocity-encoded phase-contrast imaging). The temporal resolution of CMR phase-contrast imaging even when optimized (typically 30 to 40 ms) and is much worse than the temporal resolution of TTE pulse-wave Doppler (< 10 ms). Additionally, the CMR phase-contrast imaging is an integration of segmented images captured over several cardiac cycles during a breathhold, whereas TTE pulse-wave Doppler is captured in real time over one cardiac cycle^11^.

Our study showed overall higher values in E and A using CMR compared to TTE. In this study, CMR-based trans-mitral inflow was assessed in multiple planes from the tip to the annulus of the mitral valve, and the maximum E and A values were obtained. Therefore, our CMR method appears to have captured the true maximum values of E and A waves despite the limitation of temporal resolution.

We did not optimize our CMR acquisitions for measurement of TR velocity. We felt it was technically difficult to capture the maximum flow velocity of TR because (a) of concerns that the axis of TR jet does not stay on a single plane during systole and might not be perpendicular to our mitral optimized slices; and (b) signal-to-noise ratio may be low because TR velocity is relatively low. Therefore, TTE-based TR values were used for CMR-based diastolic classification.

LAVi is also an important parameter in the assessment of LV DD^1^ because LV DD is associated with LA remodeling^12^. On TTE, the normal value of LAVi is reported as 38 ± 13 ml/m^2^ by the Simpson's method^13^, and 34 ml/m^2^ is used as the cut-off in the 2016 guideline to assess LV DD. A CMR cut-off value of LAVi to assess LV DD has not been established. Our study showed TTE measurements of LAVi were significantly smaller than CMR measurements. This finding is consistent with previous reports^6,13^. On CMR, the upper-limit of normal values have been reported as 52 ml/m^2^ for men and 53 ml/m^2^ for women^6^. Therefore, two different cut-off values were evaluated in this study to categorize CMR-based LV DD. The discrepancy of normal LAVi between the two modalities was likely due to an inaccurate derivation method of LAV on TTE since the TTE measurements were based on an estimation of LA being an ellipsoidal shape, and LAV was derived from apical 4-chamber and 2-chamber views. Whereas, our CMR measurements of LAV were derived from a contiguous short-axis cine stack of the entire LA which provides accurate volumes of structures that are model independent.

A wide spectrum of LV DD in this study cohort made it possible to compare CMR and TTE measurements throughout all the categories. Categorized analysis of parameters is particularly important in assessment of E-wave, because the velocity decreases and then increases as LV DD progresses^8^. In order to create a wide spectrum study group, we recruited consecutive patients referred to our CMR center to evaluate cardiomyopathy as well as healthy volunteers. In retrospect, we could have recruited more patients with advanced cardiomyopathies to better sample grade III diastolic abnormalities.

In this study, DD was compared between CMR and TTE using the guideline-based classification. One of the arguments evaluating DD using the current guideline is that normal or abnormal LA pressure is indirectly estimated using E/e’, indexed LAV, and maximal TR velocity. Recently, Garg et al.^14^ published an equation to estimate CMR-based pulmonary capillary wedge pressure using LAV and LV mass. CMR-based DD assessment incorporating this equation should be evaluated as a future study.

The 2016 guideline states that the standard algorithm (Fig. 4A) to assess LV DD does not apply to patients with hypertrophic cardiomyopathy^1^. We followed the separate echocardiographic algorithm to classify LV DD in patients with HCM (Fig. 4B).

## Limitations

This paper is primarily a proof-of-principle study. Our results have a possible selection bias because 22% (25 individuals) of the subjects who were initially recruited were excluded, due to irregular heart rhythm, a common limitation for cine CMR.

TTE values rather than invasive hemodynamics were used as reference trans-mitral inflow velocities. Although TTE Doppler measurements are angle dependent which can cause measurement error^15^, the Doppler technique has been validated against invasive hemodynamics and is widely accepted in clinical practice^16^. Additionally, categorization of LV DD was performed by following the diagram without considering the individual clinical picture. Accordingly, there might have been subjects who did not quite fit the guideline-based classification, and thus may have been misclassified from a clinical standpoint. However, for the purpose of this study, it was considered fair to follow a diagram without manual adjustments in order to minimize inter- and intra-observer variabilities. Lastly, TTE LAV was estimated using biplane area-length method because Simpson’s method was not available for post-processing. LAV estimated by biplane area-length method has been reported to have a strong linear relationship and agreement with those calculated by Simpson’s method^17^. Therefore we felt that biplane area-length method could be used as an alternative to Simpson’s method to estimate LAV when Simpson’s method was not available.

The proposed custom analysis software algorithm, a spatiotemporal context tracker, successfully tracked mitral valve annulus during the entire cardiac cycle, and automatically derived e’ efficiently and reliably. CMR phase-contrast imaging also provided reliable flow velocities. Classification of LV DD correlated strongly between CMR and TTE. This algorithm may make it possible to incorporate CMR-based assessment of LV DD into routine clinical practice.

## Conclusions

This study shows that LV diastolic assessment using CMR showed good concordance with the guideline-based TTE assessment, suggesting that LV diastolic dysfunction maybe classified by CMR especially when proper cut-off values specific for CMR are used. CMR is a robust imaging tool that is increasingly used for accurate diagnosis and risk stratification. LV diastolic assessment in addition to the current routine CMR practice would provide further comprehensive clinical information, and that may be important for risk stratification.

## Methods

### Patient population

This is a prospective study of patients who were referred to our CMR laboratory to evaluate cardiomyopathy between 2/2017 and 1/2018. Consecutive patients who agreed to undergo both CMR and TTE studies were recruited in this study. Both TTE and CMR studies were scheduled on the same day at National Heart, Lung, and Blood Institute in the National Institute of Health (NIH), Bethesda, MD. From the same time period, healthy volunteers (male-to-female ratio = 1) were recruited to increase the prevalence of normal diastolic function in the cohort. All the subjects gave written informed consent to participate in a clinical trial (ClinicalTrials.gov identifier: NCT00027170) that was approved by the institutional review board of NIH. Inclusion criteria for healthy volunteers were age between 18 and 40 years, asymptomatic, a Framingham risk score < 3%^18^, and Heart Score < 1%^19^. Exclusion criteria were claustrophobia, certain metallic implants, unstable angina within 48 h, hemodynamic instability, acute renal failure, and estimated glomerular filtration rate < 30 ml/min/1.73m^2^. For healthy volunteers, the following additional exclusion criteria were also applied: risk factors for coronary artery disease (hypertension, hyperlipidemia, diabetes mellitus, and smoking), history of cardiovascular disease (coronary artery disease, valvular heart disease, heart failure, arrhythmia, rheumatic heart disease, congenital heart disease, stroke, disease of the aorta or any other blood vessels), cancer, liver disease, kidney disease, lung disease, and inflammatory disease such as systematic lupus erythematosus, body mass index > 30 kg/m^2^, ventricular hypertrophy on electrocardiogram, LV ejection fraction (EF) < 50% by TTE or < 55% by CMR, and unexpected heart disease or risk factors detected as a result of this study. Patients who were unable to make appointments, severe arrhythmias, obstructive coronary artery disease, history of coronary artery bypass or percutaneous coronary intervention, congenital heart disease, or technical issues with the CMR acquisition were excluded. Patients with a regional wall motion abnormality on CMR were also excluded.

### TTE

Two-dimensional TTE was performed on commercially available echocardiography systems (Philips Healthcare, Andover, MA; or GE Healthcare, Chicago, IL) using a standard clinical protocol per American Society of Echocardiography recommendations^20^. Echocardiographic parameters were measured by two investigators (B.S. and T.A.) according to ASE guidelines^1,20^. LAV was estimated using biplane area-length method as post-processing, and then LAVi was calculated.

### CMR

#### Image acquisition

**Figure 6:**
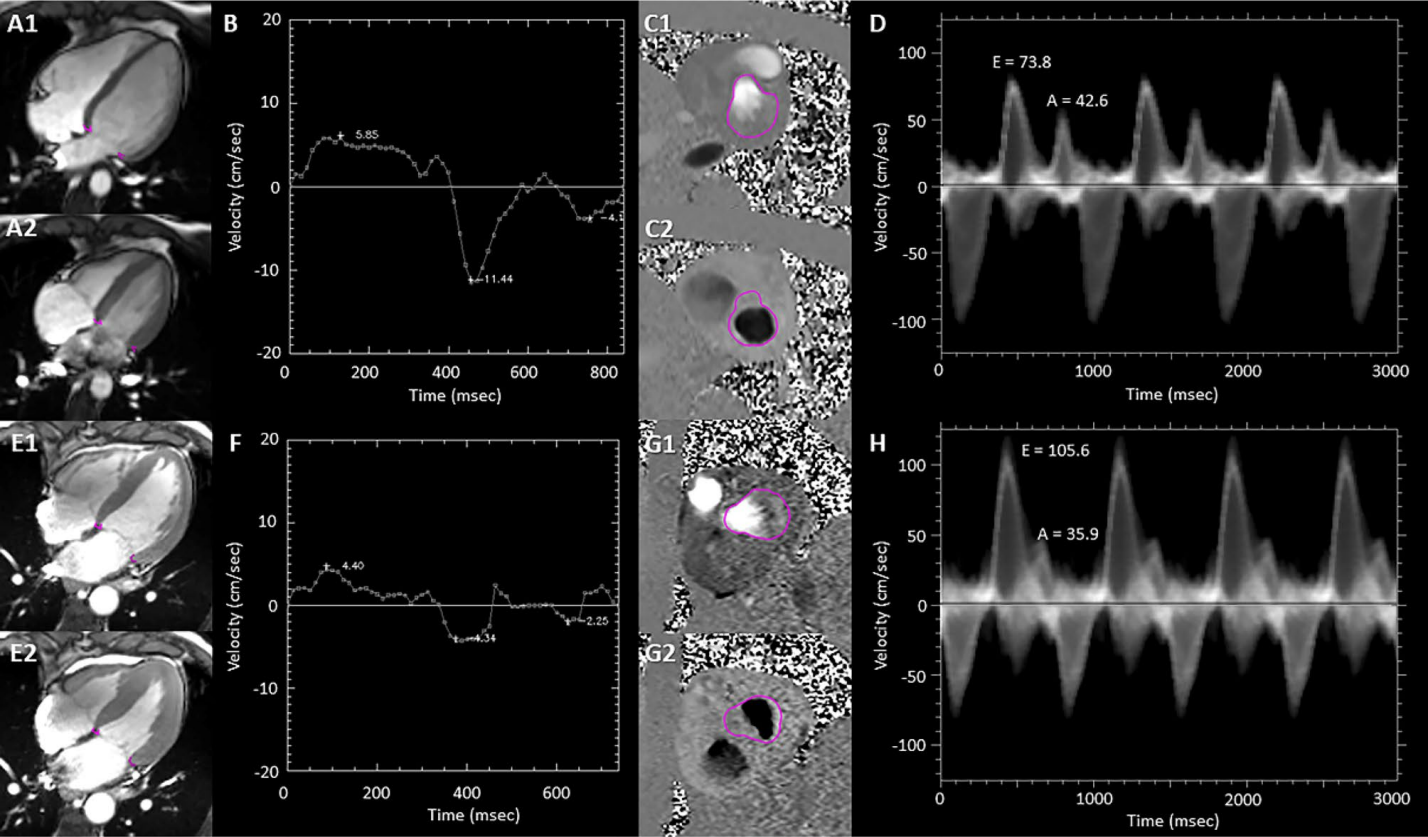
Examples of tissue tracking and phase-contrast imaging. (**A**–**D**) represent a healthy volunteer, and (**E**–**H**) represent a patient with cardiac amyloidosis. (**A**, **E**) SSFP cine imaging. On the phase 1 image (i.e. end-diastole), two anatomical locations were identified: (1) the lateral and (2) septal MVA. The MVA points were automatically tracked throughout the cardiac cycle. Regions of interest on septal and lateral MVA are shown at (A1, E1) end-diastole and (A2, E2) end-systole. (**B**, **F**) Velocity curves of the MVA points were computed and s, e’, a’ were automatically derived from the curve. (**C**, **G**) Through-plane velocity-encoded phase-contrast imaging. Flow velocity at the left ventricular outflow tract and mitral valve was traced throughout the cardiac cycle. Regions of interest are shown at (C1, G1) systolic and (C2, G2) diastolic phase. Flow velocity through the region of interest were measured throughout the cardiac cycle, and the velocity curve was derived (**D**, **H**). The peak of the early diastole (**E**) and late diastole (**A**) were manually measured. *a’* maximum mitral-annulus motion velocity during early diastole; *e’* maximum mitral-annulus motion velocity during late diastole; *LV* left ventricle; *MVA* mitral valve annulus; *RV* right ventricle; *s* maximum mitral-annulus motion velocity during systole; *SSFP* steady-state free precession.

The CMR was performed on a 3.0 Tesla magnet (Skyra, Siemens Healthineers, Erlangen, Germany) with electrocardiographic gating. Short-axis cine imaging of the entire heart was acquired using a sequence with the following characteristics: steady-state free procession (SSFP) at 30 phases of sampling per cardiac-cycle^21^. High-temporal resolution cine imaging of the 4-chamber plane was also acquired during breath-holds at 60 phases of sampling per cardiac cycle which was a double number of phase-sampling per cardiac cycle compared to our standard clinical SSFP cine imaging (Fig. 6A,E). The parameters were: TE 1.19 ms, TR 13.80 ms, flip angle 39°, 60 phases, slice thickness 8 mm, no slice gap, FOV 360 × 266 mm, Voxel size: 1.9 × 1.9 × 8.0 mm^3^, pixel bandwidth 965 Hz/pixel. Trans-mitral flow was obtained using through-plane velocity-encoded phase contrast imaging (Fig. 6C,G). The phase-contrast images were acquired on multiple parallel 6 mm thick slices from the tip of the mitral valve to the annulus (Supplemental Fig. 1). The parameters were: VENC 170 cm/s, TE 40.64 ms, TR 2.87 ms, slice thickness 6 mm, FOV 360 × 360 mm, Voxel size: 1.9 × 1.9 × 6.0 mm^3^, pixel bandwidth 491 Hz/pixel.

#### Post-processing: clinical parameters

A commercially available Leonardo workstation (Siemens Healthineers, Erlangen, Germany) was used to quantify standard clinical parameters on SSFP cine imaging acquired at 30 phases of sampling per cardiac-cycle. LAV was measured using short-axis stack of the LA. Endocardial border of the LA was delineated at the end-systole in all slices (i.e. the time point where LA is the largest during cardiac cycle), excluding the atrial appendage and the pulmonary veins. The LV volume was measured at the end-diastole (LVEDV) and end-systole (LVESV) using short-axis stack of the LV. The LV EF was calculated as a proportion of LV stroke volume (= LVEDV–LVESV) to LVEDV.

#### Post-processing: annulus motion velocity

The MVA was tracked on the 4-chamber SSFP cine images acquired at 60 phases of sampling per cardiac-cycle. On the phase 1 image, two anatomical locations were identified: 1) the lateral and 2) septal MVA. Four points were manually placed at the lateral and septal MVA (Fig. 6A,E), and were automatically tracked during the cardiac cycle using a software programmed using IDL version 8.7 (https://www.nv5geospatialsoftware.com/) (NV5 Global Inc, Broomfield, Colorado). To improve the conventional feature-tracking method based on normalized cross-correlation^5,20^, a new method integrates contextual information to a correlation filter by sampling eight neighboring sub-regions to discriminate between the target region and background is used^22^. In cases where the target contains poorly descriptive features, the context zones respond to the tracker with an adaptive penalty function to guide subsequent tracking. This method uses an online spatiotemporal context learning model to deal with anatomy deformations, illumination changes or transient noise, as is often found in dynamic cardiac series. In cases where the target contains poorly descriptive features, the context zones respond to the tracker with an adaptive penalty function to guide subsequent tracking. The quality of MVA locations across all phases was visually inspected after the automated tracking. In cases where the tracked points did not remain positioned on target structures (i.e. MVA) over the cardiac cycle, the location of tracking was manually repositioned, and then automatic MVA tracking was performed again. Velocity of each point was derived phase-by-phase, by differentiating the tracked position. On the velocity curve, maximum velocity during systole (s), e’, and late diastole (a’) were automatically derived (Fig. 6B,F).

#### Post-processing: trans-mitral valve Inflow

Trans-mitral valve inflow velocity was also analyzed using the IDL-based software. First, the mitral valve annulus and left ventricular outflow tract were traced at each phase on the through-plane velocity encoded imaging (Fig. 6C,G). Then the blood flow velocity was analyzed within the traced area throughout the cardiac cycle, and the flow velocity was displayed in shades of gray where the signal intensity represents the number of pixels with that particular velocity. The outer edge of the white outlined wave during diastole was considered to represent trans-mitral valve inflow (Fig. 6D,H). The peak of the velocity envelop at the early and late trans-mitral filling phases during diastole was measured as E and A, respectively. The flow velocity was analyzed on all the planes from tip to annulus of the mitral valve, and the peak vales of E and A among all the planes were obtained. The potential for background flow offset errors due to local non-compensated eddy currents were corrected using a static phantom imaged after the patient was out of the MRI scanner.

### Diastolic dysfunction assessment on TTE and CMR

LV DD was classified based on the 2016 guideline by American Society of Echocardiography and European Association of Cardiovascular Imaging^1^ (Fig. 4). Following the guideline^1^, the algorithm described in Fig. 4B was used in case of hypertrophic cardiomyopathy (HCM). Classification of LV DD was performed using two separate datasets (CMR-based parameters and TTE-based parameters) on each subject. E, A, lateral and septal e’, and LAVi were measured by both CMR and TTE, using the guideline cut-off values. Additional analysis was performed using CMR normal LAi values (i.e. LAVi > 52 ml/m^2^ for male, LAVi > 53 ml/m^2^ for female)^23^ for CMR-based LV DD classification. Peak velocity of tricuspid regurgitation (TR) was the only parameter that was measured only by TTE, and the TTE measurement was applied for both CMR-based and TTE-based classification.

### Statistical analysis

We analyzed the data using SAS version 9.4 (Cary, North Carolina). Subject characteristics were assessed in two groups (i.e. normal vs. abnormal) based on LV DD evaluated by TTE. Normality of the continuous variables were assessed by Kolmogorov–Smirnov test. Continuous variables were summarized as mean ± standard deviation. Categorical variables were presented as counts (percentages). Binary parameters were compared between the two groups using Fisher’s exact test. Continuous variables were compared between the two groups using Student’s t-test. CMR- and TTE-based classifications of LV DD were compared using the Fisher’s exact test. The concordance between the two modalities was evaluated as a percentage. Correlations between CMR and TTE measurements of each LV diastolic parameter were evaluated with Pearson correlation coefficient. Biases and Limits of agreement between the two modalities were evaluated using Bland–Altman plots. All statistical tests were 2-tailed, and a *p*-value < 0.05 was considered statistically significant.

### Ethics approval and consent to participate

The clinical trial was approved by the institutional review board of the National Heart, Lung, and Blood Institute (NHLBI), National Institutes of Health (NIH), Bethesda, MD. The study was performed in accordance with the Declaration of Helsinki and US Department of Health and Human Service in Federal regulations (45 CFR part 46). All the subjects gave written informed consent to participate in a clinical trial (ClinicalTrials.gov identifier: NCT00027170) that was approved by the institutional review board of the NIH.

### Supplementary Information

Supplementary Information.

## Data Availability

Data availability would have to go through a request through the authors and NIH (e.g. corresponding author). Identified and coded data cannot be provided. Only truly deidentified data can be provided.
