# Supplementary material for: CMR provides comparable measurements of diastolic function as echocardiography
Source: Sci Rep. 2024 May 22;14:11658. doi: 10.1038/s41598-024-61992-6 (PMC11111683; doi:10.1038/s41598-024-61992-6)
Supplement: Supplementary file 1 — Supplementary Information. [file 41598_2024_61992_MOESM1_ESM.pdf]

Supplemental Figure 1. Planning of 2D phase-contrast mitral valve planes. The mitral valve imaging planes were planned off the 4-chamber SSFP cine view at end-diastole with a stack of 4 or 5 slices perpendicular to the LV axis and starting at the base of the LV and progressing toward the apex with no gaps between slices (slice thickness 6 mm).

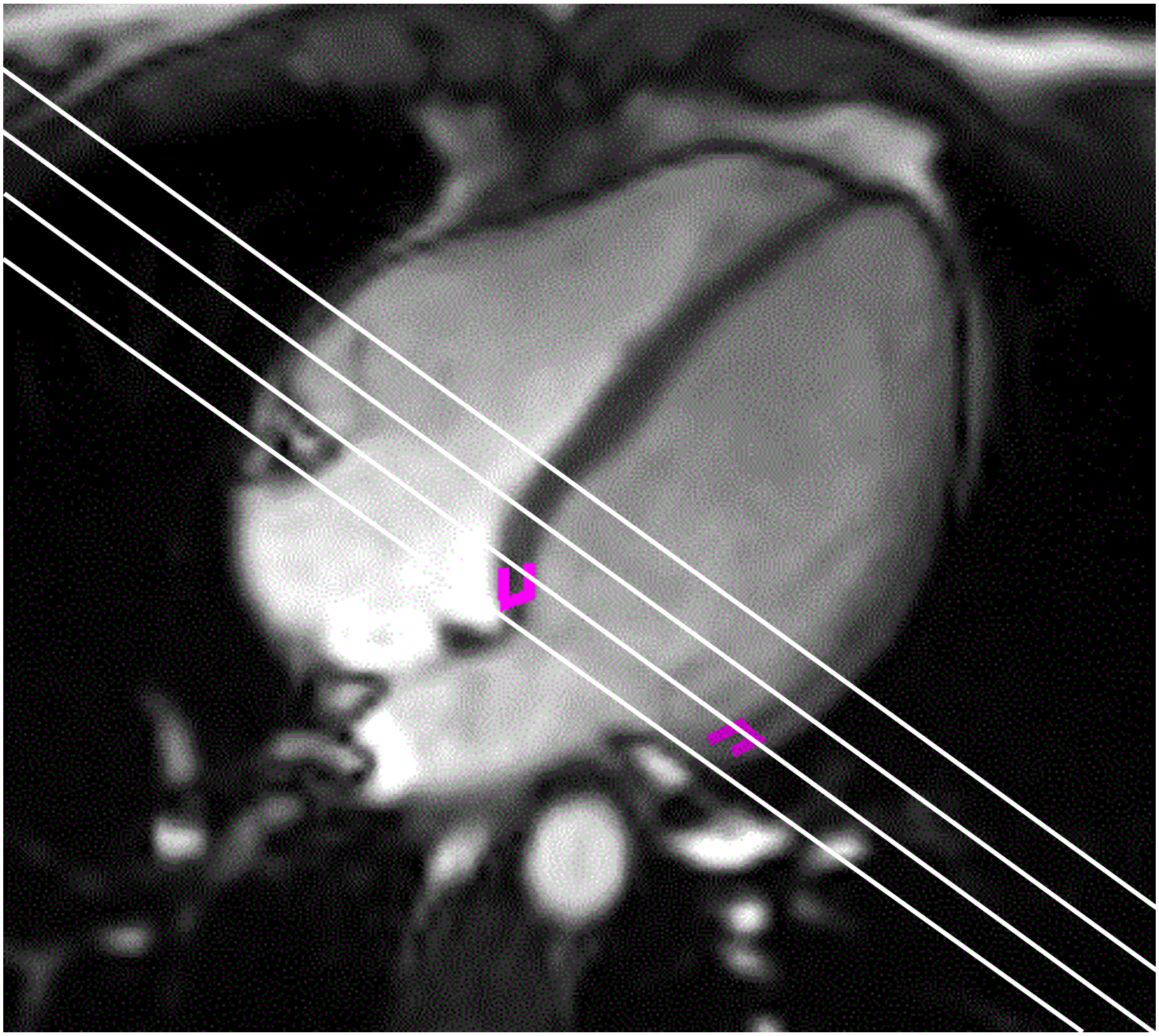

Supplemental Figure 2. Reference #10

IEEE International Symposium on Biomedical Imaging (ISBI'18)
the Omni Shoreham Hotel Washington DC, April 4 - 7, 2018

Rapid Automatic Cine MRI Myocardial Tissue Tracking Via Spatiotemporal Context Learning

Mitchel Benovoy, Bharath Sathya, Matthew Jacobs, Andrew E. Arai, Li-Yueh Hsu

National Heart, Lung and Blood Institute, National Institutes of Health, Bethesda, USA

1. INTRODUCTION

Cine cardiac magnetic resonance (CMR) imaging provides high spatial resolution and signal to noise ratio for cardiac tissue tracking. It has emerged as a promising technique for quantitative assessment of diastolic function. The derivation of myocardial velocity and displacement requires continuous tracking of anatomical structures over a cardiac cycle. We propose a fast and automatic method using a spatiotemporal context learning approach to track multichannel discriminative features of cardiac landmarks in Cine-CMR series.

2. MATERIALS AND METHODS

Eleven healthy volunteers underwent 3T CMR acquisition. Cine imaging was performed using a steady-state free precession sequence in multiplanar short-and long-axis views with electrocardiographic gating. Each study includes a 30 and 60-phase per-cardiac-cycle sampling. Our method integrates contextual information to a correlation filter tracking formulation by sampling eight neighboring sub-regions to discriminate between the target region and background. In cases where the target contains poorly descriptive features, the context zones respond with a penalty function to guide subsequent tracking. Online spatiotemporal adaptation of the learned models is used to deal with tissue deformations, illumination changes or transient occlusions, as is often found in dynamic cardiac series.

3. EVALUATION

Four anatomical points were positioned on the 4-chamber long-axis view image including the right ventricle annulus, the septal and lateral points of the atrioventricular junction, and the left ventricle apex. Tracking speed averaged 45 frames per second. For each tracked point, three velocity measures corresponding to the echocardiogram tissue doppler S/E/A waves were automatically derived from the computed velocity curves. No manual corrections were needed following automated tracking, as the tracked points remained positioned on target structures over the heart cycle. The 60-phase sampling showed a non-statistically significant trend towards higher velocities compared to the 30-phase series on all tracked points (all p-values>0.1).

4. CONCLUSION

The proposed spatiotemporal context tracker can efficiently track clinically-relevant cardiac structures to evaluate diastolic function from Cine-CMR series. No significant differences in automatically-derived velocities were observed between different sampling rates.

Supplemental Table 1. Parameters measured by CMR and TTE.

	CMR	TTE	p
E , cm/s	61.8 ± 13.9	67.9 ± 15.9	0.008
A, cm/s	47.3 ± 15.2	55.7 ± 23.2	0.005
E/A	1.5 ± 0.8	1.4 ± 0.7	0.74
septal e', cm/s	7.7 ± 3.3	8.1 ± 3.5	0.45
lateral e', cm/s	10.8 ± 4.8	11.5 ± 5.0	0.38
average e', cm/s	9.2 ± 3.9	9.8 ± 4.1	0.39
E/e'	7.9 ± 3.8	8.3 ± 4.4	0.47
LA indexed, cm^2/m^2	41.2 ± 12.3	28.9 ± 9.4	<0.0001
